# Combined computational and experimental investigation of high temperature thermodynamics and structure of cubic ZrO_2_ and HfO_2_

**DOI:** 10.1038/s41598-018-32848-7

**Published:** 2018-10-08

**Authors:** Qi-Jun Hong, Sergey V. Ushakov, Denys Kapush, Chris J. Benmore, Richard J. K. Weber, Axel van de Walle, Alexandra Navrotsky

**Affiliations:** 1 School of Engineering, Brown University, Providence, RI 02912 USA; 2 Peter A. Rock Thermochemistry Laboratory and NEAT ORU, University of California Davis, Davis, CA 95616 USA; 3 X-ray Science Division, Advanced Photon Source, Argonne National Laboratory, 9700 S. Cass Avenue, Lemont, IL 60439 USA; 4 Materials Development, Inc., 3090 Daniels Court, Arlington Heights, IL 60004 USA

## Abstract

Structure and thermodynamics of pure cubic ZrO_2_ and HfO_2_ were studied computationally and experimentally from their tetragonal to cubic transition temperatures (2311 and 2530 °C) to their melting points (2710 and 2800 °C). Computations were performed using automated *ab initio* molecular dynamics techniques. High temperature synchrotron X-ray diffraction on laser heated aerodynamically levitated samples provided experimental data on volume change during tetragonal-to-cubic phase transformation (0.55 ± 0.09% for ZrO_2_ and 0.87 ± 0.08% for HfO_2_), density and thermal expansion. Fusion enthalpies were measured using drop and catch calorimetry on laser heated levitated samples as 55 ± 7 kJ/mol for ZrO_2_ and 61 ± 10 kJ/mol for HfO_2_, compared with 54 ± 2 and 52 ± 2 kJ/mol from computation. Volumetric thermal expansion for cubic ZrO_2_ and HfO_2_ are similar and reach (4 ± 1)·10^−5^/K from experiment and (5 ± 1)·10^−5^/K from computation. An agreement with experiment renders confidence in values obtained exclusively from computation: namely heat capacity of cubic HfO_2_ and ZrO_2_, volume change on melting, and thermal expansion of the liquid to 3127 °C. Computed oxygen diffusion coefficients indicate that above 2400 °C pure ZrO_2_ is an excellent oxygen conductor, perhaps even better than YSZ.

## Introduction

Hafnium and zirconium oxides are indispensable constituents for development of the formulations for structural ceramics^1^, thermal barrier coatings^2^, high temperature refractories^3^ and for nuclear applications, such as matrices for fission and transmutation and sacrificial materials for core catchers for next generation nuclear reactors^4^. ZrO_2_ and HfO_2_ are isostructural and exhibit monoclinic-tetragonal-cubic transformations before melting at 2710 and 2800 °C, respectively. Thermodynamic assessments for pure oxides to the melting temperatures are required for prediction of phase composition, stability, and microstructure in multicomponent systems using Calphad type^5^ approaches, which have proven to be extremely useful in metallurgy and ceramics.

The latest review of experimental data and assessment of the Gibbs free energy functions for all HfO_2_ and ZrO_2_ phases was performed by Wang, Zinkevich and Aldinger in 2006^6^ (referred further as the WZA assessment). It was adopted by most researchers for Calphad modeling for ZrO_2_- and HfO_2_- containing systems^2,7^. A plethora of computational and experimental investigations has been devoted to the thermodynamics of monoclinic and tetragonal phases^8,9^, and the structure of the liquid was studied experimentally and computationally^10,11^. However, for the cubic phases we only know unambiguously that they are stable for a few hundred degrees before melting and have unit cell parameters somewhere between 5.1 and 5.3 Å^12^. Measurements of enthalpy increments for cubic ZrO_2_ and HfO_2_ phases were performed by Pears *et al*. in 1963^13^. However, their samples were exposed to carbon vapor in a graphite furnace and their data were not used in the WZA assessment^6^. The value for ZrO_2_ fusion enthalpy (87 kJ/mol) reported in the JANAF tables^14^ and used by WZA^6^ can be traced^15^ to an assessment made by Kelley in 1936^16^ based on the slope of the solidus in early ZrO_2_-SiO_2_ and ZrO_2_-MgO phase diagrams. Thus, we conclude that no direct experimental measurements of fusion enthalpy for ZrO_2_ and HfO_2_ have been performed to date.

In this work, we sought to fill this gap in the available data by measuring and computing the fusion enthalpies of ZrO_2_ and HfO_2_. The combination of experimental and computational method offers a unique opportunity for corroboration that is essential given the challenges associated with each approaches. On the experimental side, the difficulties lie in thermal gradients unavoidable in conditions of uniaxial laser heating of aerodynamically levitated samples used for calorimetry^17,18^ and X-ray diffraction^19–21^. On the computational side, the difficulties reside in reaching sufficiently large system sizes and sufficiently long simulation times while still using accurate electronic structure calculations as well as ensuring proper modeling of all forms of excited states (defect formation and diffusion, potential anharmonic phonons and electron excitations). Thermal expansions of cubic ZrO_2_ and HfO_2_ and volume change during the transition from the tetragonal phase were measured by high temperature X-ray diffraction experiments. The agreement between computed and measured values for fusion enthalpies and for thermal expansion supports the validity of the heat capacities, diffusion coefficients, and volume change upon melting obtained from the computation.

## Results and Discussion

**Figure 1:**
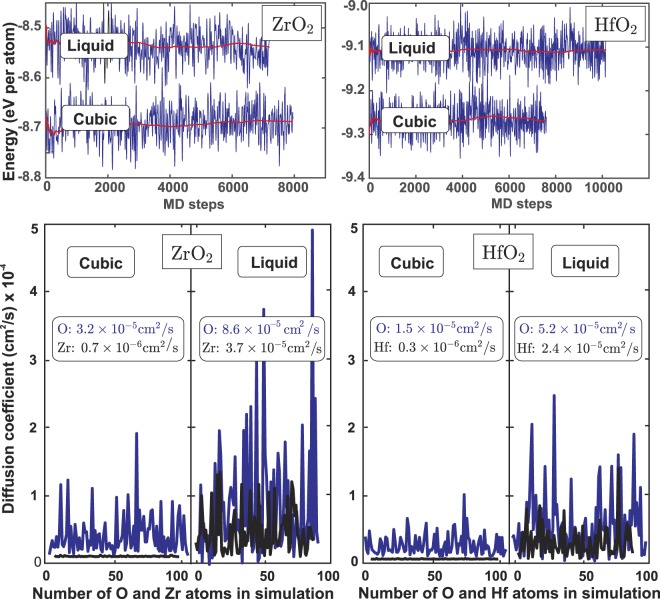
Top: Energy per atom in MD simulations for ZrO_2_ and HfO_2_ in solid cubic fluorite phase at 2727 °C and in liquid state at 2827 °C. Bottom: diffusion coefficients for O, Zr and Hf atoms in cubic and liquid phases obtained from the last 12 ps for the cubic and 29 ps for the liquid MD trajectories.

A summary of the results of *ab initio* computations is presented in Table 1 and Fig. 1. Results from high temperature X-ray diffraction are tabulated in Supplementary Information. Below, the thermodynamic data for cubic ZrO_2_ and HfO_2_ from computation and experiment are discussed together in the same order as in Tables 2 and 3 and are compared with literature values.

**Table 1:** Results of *ab initio* MD computations for ZrO_2_ and HfO_2_ on 270 atoms.

	*T*, °C	CPU, Hours	MD length, ps	Volume, Å^3^/atom	Energy, eV/atom	HSE *P* correction, kBar	HSE *E* correction, eV/atom	*a*, Å	Density, g·cm^−3^
Cubic ZrO_2_	2327	10600	15	12.32 (2)	−8.829 (5)	−52.02	−1.97	5.288 (2)	5.54 (1)
	2527	26000	34	12.43 (2)	−8.761 (4)	−51.19	−1.97	5.303 (2)	5.49 (1)
	2627	26500	34	12.50 (2)	−8.720 (4)	−50.83	−1.97	5.313 (2)	5.46 (1)
	2727	11200	14	12.54 (2)	−8.687 (4)	−51.08	−1.96	5.320 (2)	5.44 (1)
Liquid ZrO_2_	2827	29300	31	14.03 (5)	−8.490 (4)	−44.45	−1.93		4.86 (2)
	2927	29300	31	14.16 (4)	−8.456 (4)	−43.91	−1.92		4.82 (1)
	3127	29600	28	14.39 (4)	−8.383 (4)	−43.45	−1.92		4.74 (1)
Cubic HfO_2_	2527	19200	62	11.96 (1)	−9.346 (4)	−59.05	−1.98	5.235 (2)	9.74 (1)
	2627	19200	59	12.00 (1)	−9.306 (4)	−59.34	−1.98	5.242 (2)	9.71 (1)
	2727	7800	23	12.08 (2)	−9.264 (6)	−58.84	−1.98	5.253 (2)	9.65 (1)
Liquid HfO_2_	2827	21500	56	13.35 (5)	−9.068 (4)	−51.89	−1.95		8.73 (3)
	2927	21800	56	13.40 (5)	−9.038 (4)	−51.46	−1.94		8.69 (3)
	3127	22800	55	13.66 (4)	−8.963 (7)	−51.36	−1.94		8.53 (2)

**Table 2:** Thermodynamic data for cubic and liquid ZrO_2_.

Phase/Property	Value	Method	Reference
**Cubic ZrO**_**2**_			
*T* (T-C) trs, °C	2311	Experimental best value†	WZA 2006^6^
T-C Δ*V*,%	0.55 ± 0.09	XRD Experiment	This work
Density, g/cm^3^	5.61–5.53	XRD at (2311–2710 °C)	This work
	5.54–5.44	*Ab initio* MD at 2327–2727 °C	This work
*C*_p_, J/mol/K	111 ± 7	*Ab initio* MD at 2327–2727 °C	This work
Linear TEC, *α*, K^−1^	(1.2 ± 0.3)·10^−5^	HT XRD at (2311–2710 °C)	This work
Vol. TEC, *α*_v_, K^−1^	(3.7 ± 0.9)·10^−5^	HT XRD at (2311–2710 °C)	This work
	(4.8 ± 0.7)·10^−5^	*Ab initio* MD at 2527–2727 °C	This work
*T*_mlt_, °C	2710	Experimental best value^†^	WZA 2006^6^
Δ*V* on melting, %	11 ± 2	Ab initio MD	This work
Δ*H*_fus_, kJ/mol	87	Assessment	Kelley 1936^16^
	55 ± 7	DnC experiment	This work
	54 ± 2	*Ab initio* MD	This work
	26–49	Classic MD	Kim *et al*.^36^
Δ*S*_fus_, J/mol/K	29	Assessed Δ*H*/*T*_m_ (K)	WZA 2006^6^
	18	Experiment Δ*H*/T_m_ (K)	This work
	17	*Ab initio* MD Δ*H*/*T*_m_ (K)	This work
**Liquid ZrO**_**2**_			
Density Liq, g/cm^3^	4.86–4.74	*Ab initio* MD at 2827–3127 °C	This work
	5.1–4.9	Experiment at 2710–3000 °C	Kohara *et al*.^11^
*C*_p_ Liq, J/mol/K	116 ± 25	*Ab initio* MD at 2827–3127 °C	This work
	100	Classic MD	Kim *et al*.^36^
Vol. TEC, *α*_v_, K^−1^	(8.7 ± 0.2)·10^−5^	*Ab initio* MD at 2827–3127 °C	This work

^†^Best values for ZrO_2_ tetragonal–cubic (T-C) transition and melting from WZA assessment of experimental results (2311 and 2710 °C) were used for temperature calibration in diffraction experiments in this work. (TEC: Thermal Expansion Coefficient, Vol.: Volumetric).

**Table 3:** Thermodynamic data for cubic and liquid HfO_2_.

Phase/Property	Value	Method	Reference
**Cubic HfO**_**2**_			
*T* (T-C) trs, °C	2530	Experimental best value^†^	WZA 2006^6^
T-C Δ*V*, %	0.87 ± 0.08	HT XRD Experiment^†^	This work
Density, g/cm^3^	9.68–9.58	XRD at (2530–2800 °C)^†^	This work
	9.74–9.65	*Ab initio* MD at 2527–2727 °C	This work
*C*_p_, J/mol/K	126 ± 4	*Ab initio* MD at 2527–2727 °C	This work
Linear TEC, *α*, K^−1^	(1.3 ± 0.4) ·10^−5^	HT XRD at (2530–2800 °C)^†^	This work
Vol. TEC, *α*_v_, K^−1^	(4 ± 1) 10^−5^	HT XRD at (2311–2710 °C)^†^	This work
	(5.0 ± 0.7) ·10^−5^	*Ab initio* MD at 2527–2727 °C	This work
*T*_mlt_, °C	2800	Experiment best value	WZA 2006^6^
Δ*V* on melting, %	10 ± 2	*Ab initio* MD	This work
Δ*H*_fus_, kJ/mol	89.6	Assessed Δ*S*·*T*_m_	WZA 2006^6^
	61 ± 10	DnC experiment	This work
	52 ± 2	*Ab initio* MD	This work
Δ*S*_fus_, J/mol/K	29	Assessed from ZrO_2_ data	WZA 2006^6^
	20	Experiment Δ*H*/*T*_m_ (K)	This work
	17	*Ab initio* MD Δ*H*/*T*_m_ (K)	This work
**Liquid HfO**_**2**_			
Density Liq, g/cm^3^	8.73–8.53	*Ab initio* MD at 2827–3127 °C	This work
	8.16	PDF experiment	Gallington 2017^10^
*C*_p_ Liq, J/mol/K	109 ± 15	*Ab initio* MD at 2727–3127 °C	This work
Vol. TEC, *α*_v_, K^−1^	(8 ± 1)·10^−5^	*Ab initio* MD at 2827–3127 °C	This work

^†^Best values for HfO_2_ tetragonal–cubic (T-C) transition and melting from WZA 06 assessment of experimental results (2530 and 2800 °C) were used for temperature calibration in diffraction experiments in this work. (TEC: Thermal Expansion Coefficient, Vol.: Volumetric).

### Tetragonal - cubic transition and thermal expansion of cubic phases

**Figure 2:**
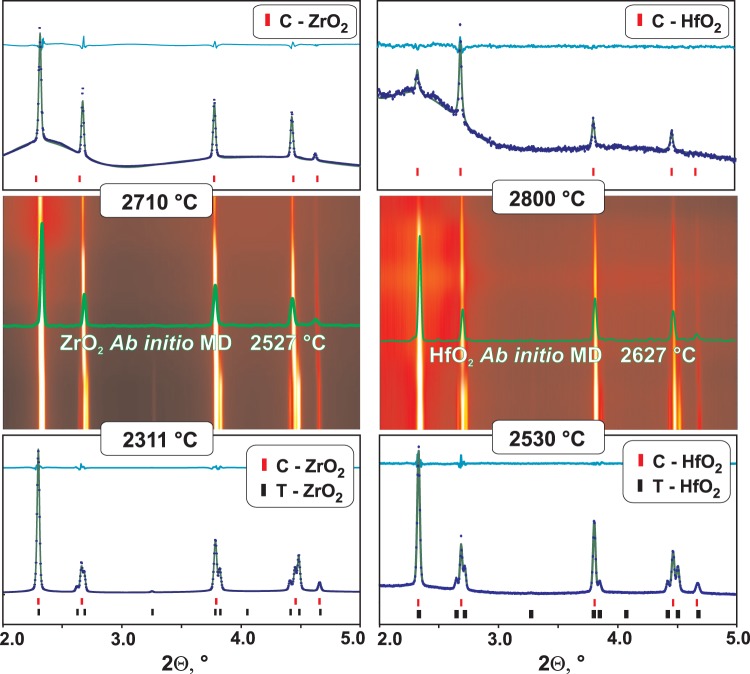
Center: contour plots of X-ray diffraction (XRD) patterns (λ = 0.12359 Å) with cubic ZrO_2_ and HfO_2_ from tetragonal-to-cubic transition to melting onset. The patterns obtained from *ab initio* MD simulations are included for comparison. Top and bottom: Pawley refinements of XRD patterns of cubic ZrO_2_ and HfO_2_ in the presence of melt and tetragonal phase (experimental data points, modeled pattern and difference curve). See Supplementary Information for refinement results for all patterns depicted in contour plots.

Temperatures for tetragonal-cubic transition and melting points for ZrO_2_ and HfO_2_ were accepted from the WZA^6^ assessment and were used in this work for the evaluation of the temperature of the diffracted volume of the laser heated samples. Cubic ZrO_2_ and HfO_2_ have a fluorite structure with space group Fm3m and 4 formula units per cell (Z = 4). Besides the mineral fluorite (CaF_2_), which gives the name for the structure type, natural and synthetic uraninite (UO_2_), thorianite (ThO_2_), and cerianite (CeO_2_) are found in this structure. Thermophysical properties of UO_2_ and ThO_2_ above 2000 °C were studied extensively for nuclear reactors safety assessments^22,23^, and a comparison of the high temperature structures for UO_2_ with ZrO_2_ and HfO_2_ from this work is given at the end of this paper. In the tetragonal (P4_2_/mmc, Z = 2) and cubic phases, Zr and Hf are coordinated by eight oxygen atoms, but in the monoclinic structure (*P*2_1_/c), stable at room temperature, the cation coordination is 7. Unit cell parameters of the tetragonal and cubic ZrO_2_ and HfO_2_ at transition temperatures were refined from XRD patterns containing both phases (Fig. 2), giving volume change upon transition. There are a number of values in the ICSD database^24^ for volumes of stable and metastable tetragonal ZrO_2_ at temperatures below 1627 °C (see Supplementary Information). Our value for the volume of tetragonal ZrO_2_ at the transition temperature is consistent with the trend of close to linear volume expansion of the tetragonal phase, yielding an average value for of volumetric thermal expansion (α_v_) of 3.9·10^−5^ K^−1^ in the 300–2311 °C range.

At the transition temperatures, refined unit cells (*a* × *c*) for tetragonal ZrO_2_ and HfO_2_ are 3.690 × 5.337 Å and 3.669 × 5.327 Å, respectively. The variation in cell parameters from Pawley refinements of individual patterns on the same sample is within ±0.001 Å (Tables S2 and S3). Based on refinements from different beads, accuracy is estimated to be within ±0.003 Å, and this uncertainty has been propagated to experimental density and thermal expansion values in Tables 2 and 3. Both ZrO_2_ and HfO_2_ show less than 1% volume increase during the tetragonal to cubic transformation. From X-ray diffraction, unit cell parameters for cubic ZrO_2_ increases from 5.265 to 5.291 Å from the tetragonal-cubic transformation temperature to the melting temperature. The corresponding values for HfO_2_ are 5.246 and 5.265 Å. The cell parameters from *ab initio* MD computations (Table 1) show good agreement with experiment with differences less than 0.5%. After propagation of uncertainties, the experimental values for volumetric thermal expansion are in good agreement with computations and within 4 (±1)·10^−5^ K^−1^ for both cubic ZrO_2_ and HfO_2_ in their stability range (Tables 2 and 3).

We did not locate any previous reports on experimental or computational values for the thermal expansion. There are few reported values for the cell parameters of high temperature cubic ZrO_2_ and HfO_2_ and all of them were measured in vacuum and thus on possibly somewhat reduced samples. In fact, even though cubic ZrO_2_ was assumed in the early phase diagrams by Kelley^16^ in 1936 for the assessment of the fusion enthalpy, the existence of pure cubic phases at high temperature was still questioned in 1962^25^, due to the lack of structural data in oxidizing conditions. Boganov *et al*.^26^ studied high temperature transformations in ZrO_2_ and HfO_2_ in a vacuum of 5·10^−6^ Torr with heating by the electron beam and reported the unit cell parameter for ZrO_2_ as 5.256(3) Å at 2330 °C and for HfO_2_ as 5.30(1) Å at ~2700–2750 °C. The latter value for cubic HfO_2_ was cited in reviews by Glushkova^27^ and Wang^12^. Considering experimental conditions, these values probably refer to oxygen deficient cubic ZrO_2−x_ and HfO_2−x_, known to exist in Zr-O and Hf-O systems^28^, and thus the differences with the results of our work are expected. Passerini^29^ derived room temperature cell parameters for cubic ZrO_2_ and HfO_2_ as 5.065 Å and 5.115 Å by extrapolation from their fluorite solid solutions with CeO_2_. Combining his values with cell parameters before melting from this work (5.291 and 5.265 Å) gives an average volumetric thermal expansion from room temperature to the melting points of ~5·10^−5^ K^−1^ for ZrO_2_ and ~3·10^−5^ K^−1^ for HfO_2._

### Volume change upon melting, density and thermal expansion of liquid ZrO_2_ and HfO_2_

**Figure 3:**
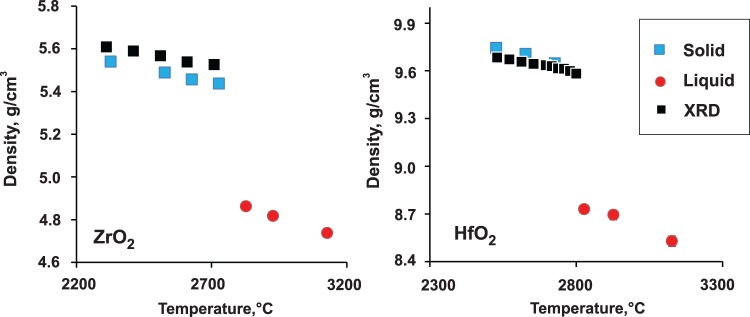
Density change on melting of cubic ZrO_2_ and HfO_2_ from *ab initio* MD computations with overlayed experimental results from high temperature X-ray diffraction (XRD). Uncertainties from computation and experiment are smaller than the symbol size. (The computational results are in Table 1, the results of Pawley refinement of XRD patterns are provided in Supplementary Information).

At 25 °C, our computation gives volume of monoclinic ZrO_2_ and HfO_2_ as 35.22 and 34.11 Å^3^ per formula unit, respectively. This compares well with experimental values of 35.15 and 34.57 Å^3^ per formula unit by Hann^30^. The density change of cubic ZrO_2_ and HfO_2_ with temperature from high temperature XRD data is shown in Tables 2 and 3 and Fig. 3 and compared with the results from computations. The good agreement allows us to rely on a*b initio* MD results for volume change upon melting as well as density and thermal expansion of the liquid phases. ZrO_2_ and HfO_2_ show similar expansion upon melting, 11 ± 2% and 10 ± 2%, respectively. For the temperature range sampled by computation, volumetric thermal expansions of liquid ZrO_2_ and HfO_2_ fall within (8 ± 1)·10^−5^ – twice that for the cubic phase. Despite known biases in the lattice parameters calculated via DFT methods^31^, calculated volume changes tend to be much more accurate, due to systematic error cancellations.

To the best of our knowledge, the volume change on melting has not been previously quantified. There are some published values for the density of liquid phases, since it has to be measured or refined for the analysis of the liquid structure by the pair distribution function (PDF) method^10,11^. The density of liquid ZrO_2_ was recently measured by Kohara *et al*.^11^ from the dimensions of aerodynamically levitated liquid ZrO_2_ spheroids. His values, 5.1–4.9 g/cm^3^ for in 2710–3000 °C, are in good agreement with our results 4.86–4.74 g/cm^3^ at 2827–3127 °C. The density of liquid HfO_2_ was refined from PDF measurements by Gallington *et al*.^10^ as 8.16 g/cm^3^, compared with 8.73–8.53 g/cm^3^ at 2827–3127 °C from our computations.

### Enthalpy and entropy of fusion

**Figure 4:**
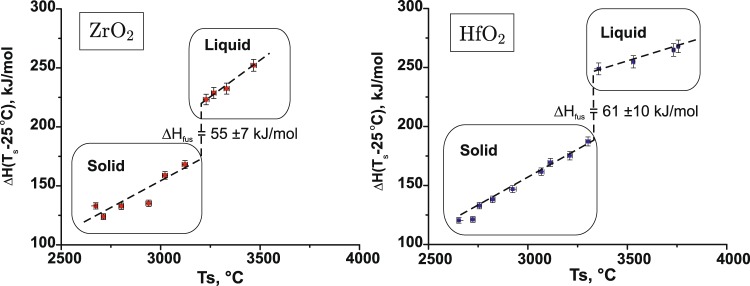
Fusion enthalpy from drop and catch calorimetry on ZrO_2_ and HfO_2_ in argon flow. T_s_ – surface temperature before the drop measured by spectropyrometer.

*Ab initio* MD computations resulted in values for fusion enthalpies of (Δ*H*_fus_) 54 ± 2 kJ/mol for ZrO_2_ and 52 ± 2 kJ/mol for HfO_2_. They agree, within experimental uncertainties, with values from the drop and catch calorimetry from samples levitated in argon flow (Fig. 4). It must be noted, however, that calorimetry experiments performed in oxygen flow did not provide a well defined step for HfO_2_ fusion and resulted in a larger value for ZrO_2_ (see Supplementary Information). This cannot be related to the sample reduction during levitation in Ar flow, as the calorimeter is not enclosed in the chamber, there is enough air entering in the levitation stream through turbulence to prevent ZrO_2_ and HfO_2_ reduction, and the samples were white in color after the drop experiments in Ar. We attribute observed differences to possible oxygen dissolution in ZrO_2_ and HfO_2_ melts, an effect previously observed by Coutures^32^ in a number of oxide melts. The possibity of oxygen dissolution in molten ZrO_2_ has profound implications for Zr-O phase equilibria at high oxygen fugacities and deserves a separate in-depth study.

In most of the assessments of the thermodynamic functions of cubic ZrO_2_, reviewed in detail by Wang *et al*.^28^, fusion enthalpy was kept fixed to the value 87 kJ/mol from JANAF tables^14^; it was optimized to 68 kJ/mol in Chen *et al.’s*^33^ assessment for the ZrO_2_-YO_1.5_ system, while Chevalier *et al*.^34^ obtained 90 kJ/mol. The value 87 kJ/mol was also accepted in the WZA^6^ assessment and used for the calculation of ZrO_2_ fusion entropy (Δ*S*_fus_ = Δ*H*_fus_/*T*_m_ (K) = 29 J/mol/K). The fusion enthalpy for HfO_2_ was then estimated^6^ from the melting temperature based on the assumption that it has the same fusion entropy as ZrO_2_. It must be noted that the widely used value for Δ*H*_fus_ ZrO_2_ from the JANAF Thermochemical tables^14^ can be traced back to the 1951 data compilation by Wagman *et al*.^15^ The same value is reported in Glushko’s^35^ compendium of thermodynamic properties of individual substances with reference to Kelley 1936^16^ who calculated heats of fusions from available freezing point data of the binary systems ZrO_2_ with SiO_2_ and MgO. This approach is limited by a lack of information concerning activities in the liquid and solid solutions and by its reliance on the accuracy of the phase diagram determination. It is impressive that Kelley’s^16^ early assessment held for 80 years without any challenge.

The values computed for the fusion enthalpies of ZrO_2_ and HfO_2_ in this work (54 ± 2 and 52 ± 2 kJ/mol) are substantially lower than Kelley’s 87 kJ/mol value included in JANAF tables^14^ and used in the most current thermodynamic assessments^6,36^. They agree, within experimental uncertainties, with our drop and catch calorimetry measurements. Using accepted melting temperatures, the entropy of fusion for ZrO_2_ and HfO_2_ calculated as 17 J/mol/K, which is substantially lower than Δ*S*_fus_ 29 J/mol/K value obtained from Kelley’s estimate and used in the WZA^6^ assessment. While our experiments were in progress, a Δ*H*_fus_ for ZrO_2_ was reported by Kim *et al*.^36^ as 26–49 kJ/mol from classical MD simulations based on interatomic potentials. We did not locate any reports on the computation of the fusion enthalpy of HfO_2_.

### Heat capacities

As previously discussed^17^, drop and catch calorimetry cannot yet provide reasonably accurate values for the heat capacity due to differences in heat loss by radiation from different temperatures. The heat capacities of cubic ZrO_2_ and HfO_2_ obtained from *ab initio* MD computations are 111 ± 7 J/mol/K and 126 ± 4 J/mol/K, respectively. The values computed for liquid ZrO_2_ and HfO_2_ at the modeled temperatures are close to the values for the cubic phase when uncertainties are taken into account (see Tables 2 and 3). Five different thermodynamic models have been proposed in recent years to model cubic zirconia and the liquid phase in assessments of the Zr-O system^28^. In the absence of data on the thermodynamics of cubic ZrO_2,_ they relied mostly on the reproduction of ZrO_2−x_ – Zr(O) and ZrO_2−x_ – liquid phase boundaries^37^. Heat capacities of cubic and liquid ZrO_2_ calculated from different assessments are reviewed by Wang *et al*.^28^ and for most models, they are in the range of 75–90 J/mol/K for cubic ZrO_2_ and 80–100 J/mol/K for the liquid, below 3727 °C. Our computed values are close to 15R (where R is the gas constant) and substantially higher than those used in the assessments and higher than the 9R high temperature limit of Dulong and Petit for the contribution of lattice vibration. With the exception of UO_2_, which melts at 2874 °C, there are no experimental data for the heat capacity of fluorite type oxides melting in a comparable temperature range. Ronchi *et al*.^38,39^ reported measurements of heat capacity for UO_2_ from 1600 °C to 5000 °C using a custom-designed laser flash instrumentation. Their results indicate that UO_2_ heat capacity exceeds 20R before melting, decreases to 15R after melting and decreases further to the 9 R limit only above 4000 °C. The excess heat capacity in UO_2_ at high temperature is attributed to both electronic transitions and to disorder on the oxygen sublattice. The latter is also known as the Bredig^40^ transition, which is common among fluorite halides and oxides above 0.8·T_m_. Clearly, the high temperature heat capacity needs further study.

### Structure of cubic ZrO_2_ and HfO_2_

In the fluorite structure (*Fm*3*m*) all atoms are located on special equivalent positions – cations on 4(a) at the origin and anions on 8(c) at ¼, ¼, ¼. In stoichiometric HfO_2_ and ZrO_2_, both sites are fully occupied, and the structure is uniquely defined by its unit cell parameter and the atomic displacement parameters for Zr or Hf and O atoms. In *ab initio* MD computations, HfO_2_ and ZrO_2_ stoichiometries were preset by the number of atoms in the simulation. High temperature diffraction experiments were performed in oxygen flow, and the samples remained white in color after melting, but the possibility of thermally induced oxygen defects in the cubic phases cannot be ruled out. The quality of the diffraction data did not allow refinement of oxygen occupancies due to the strong correlation with atomic displacement parameters (ADP). Isotropic ADPs were refined from selected XRD patterns as mean square displacement amplitude *U*_iso_ (Å^2^) and estimated from snapshots of MD trajectories (see Supplementary Information). *U*_iso_ for Zr and Hf and for oxygen in HfO_2_ are in agreement from XRD and MD and vary within 0.03–0.05 Å^2^ for cations and increase from 0.03 to 0.07 for oxygen in hafnia. Both experiment and computation indicate larger displacement amplitudes for oxygen in zirconia: Oxygen *U*_iso_ was determined to range from 0.08 to 0.15 Å^2^ from XRD data, while MD results indicate even larger amplitudes: from 0.19 to 0.29 Å^2^. It must be noted that, in our related experimental and computational study of lanthanum zirconate^41^, we also found good agreement in thermal expansion, but higher O displacement amplitudes from *ab initio* MD compared to those inferred from high temperature XRD data.

### Atomic diffusion in cubic and liquid ZrO_2_ and HfO_2_

Diffusion rates for Zr, Hf and O in cubic phases and in the liquid obtained from simulations for cubic and liquid phases are shown in Fig. 1. The proximity of diffusion rates of oxygen in cubic and liquid phases explains high heat capacity in fluorite phase and suggests that the notion of “oxygen sublattice melting” is an accurate description of the Bredig transition. Note that tetragonal – cubic transformation in ZrO_2_ and HfO_2_ was suggested to be a second order transition^6^ and occurs shortly after exceeding 80% of the melting temperature threshold for the Bredig transition in fluorite structure.

**Figure 5:**
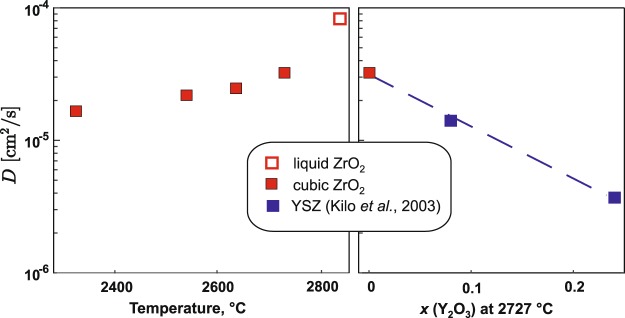
Oxygen diffusion coefficients in pure zirconia computed in this work compare with values for YSZ with 8% and 24% Y_2_O_3_ extrapolated to 2727 °C from Kilo *et al*.^41^.

Diffusion coefficients were calculated from the MD trajectories, according to equation^42^

$$\langle {r}_{i}^{2}(t)\rangle =\frac{1}{N}\sum _{i=1}^{N}{[{r}_{i}(t)-{r}_{i}(0)]}^{2}=6Dt,$$

where *D* is diffusion coefficient, *t* is time, *r* is atomic position and *N* is number of atoms. Temperature dependent diffusion coefficients are summarized in the Supplementary Information. Our computations show negligible Zr and Hf diffusion rates in stability range of cubic phases: within 0.4–0.7·10^−6^ cm^2^/s for Zr and 0.1–0.3·10^−6^ cm^2^/s for Hf. Oxygen diffusion coefficients above tetragonal-cubic transition temperatures are an order of magnitude higher than those for cations, which suggests significant oxygen diffusion. Notably, modeling cubic HfO_2_ 200 °C below its stability field does not show noticeable difference in Hf diffusion coefficient but show 10 fold decrease in oxygen diffusion (Table S5). Kilo *et al*.^42^ reported MD computations of oxygen diffusion in YSZ with 8 and 24 mol % Y_2_O_3_ from 400 to 1600 °C. Figure 5 show oxygen diffusion coefficients in ZrO_2_ as a function of temperature and as a function of Y content using values extrapolated from Kilo’s study. It is remarkable that oxygen diffusion in pure ZrO_2_ is higher than in YSZ at any temperature and linear dependence on Y content is observed.

### Comparison with UO_2_, ThO_2_, and fluorite-related bixbyite and pyrochlore structures

Thoria and urania both retain a fluorite structure from ambient temperature to their respective melting points (2874 and 3367 °C). Both oxides are believed to exhibit Bredig transitons above 0.8·*T*_m_. ThO_2_ is the only known Th oxide and expected to be more similar to cubic HfO_2_ and ZrO_2_ than UO_2_ which is known to exhibit electronic transitions and substantial hypo- and hyperstoichiometry ranges with a fraction of U going into trivalent or pentavalent states. For UO_2_ at above 0.8·*T*_m_ the linear thermal expansion increases^23^ to 30·10^−6^ K^−1^, Oxygen *U*_iso_ to 0.12 Å^2^ ^22^, and melting is accompanied by 10 ± 1% volume increase^43^. The data for ThO_2_ at above 0.8·*T*_m_ are scarce, hence the thermal expansion at the melting point was extrapolated^44^ to be 14·10^−6^ K^−1^, and Oxygen *U*_iso_ follows the trend for UO_2_ ^22^, but was not measured above 0.8·*T*_m_.

Yb_2_O_3_ and Lu_2_O_3_ melt at 2435 and 2490 °C, respectively, and are stable in bixbyite or C-type structure (Ia3, Z = 16), which is often described as a derivative of a defected fluorite structure having ordered vacancies. Their linear thermal expansion was studied^21^ both in argon and oxygen and was reported to not exceed 8.5·10^−6^ K^−1^ with *U*_iso_ values for Yb and Lu below 0.05 Å^2^ up to the melting temperature and *U*_iso_ values for O less than 0.07 Å^2^. Lanthanum zirconate (La_2_Zr_2_O_7_ or LZ) is an example of compound stable up to the melting temperature in the pyrochlore (Fd3m, *Z* = 8) structure, which is often described as a defected fluorite structure with ordering of both cations and oxygen vacancies. Neutron diffraction in Ar atmosphere indicates that it does not display an anomalous thermal expansion or oxygen mobility indicative of a Bredig transition. The linear thermal expansion of LZ was reported as ~7·10^−6^ K^−1^ from above 1650 °C to the melting temperature of 2300 °C, with *U*_iso_ values for O and La not exceeding 0.07 Å^2^ and that of Zr remaining below 0.03 Å^2^ ^20^.

The linear thermal expansion of fluorite ZrO_2_ and HfO_2_ (~12·10^−6^ K^−1^) are substantially higher than for LZ and C-type Yb_2_O_3_ and Lu_2_O_3_ (<8.5·10^−6^ K^−1^), and lower than that observed before melting in UO_2_ (~30·10^−6^ K^−1^) and defect fluorite YSZ (~25·10^−6^ K^−1^)^20^, and similar to the value accepted^44^ for ThO_2_ (14·10^−6^ K^−1^). Notably, despite apparently higher oxygen mobility in ZrO_2_ than in HfO_2_, their molar heat capacities and volume changes on melting are the same within uncertainties. Computational and experimental results suggest dynamic disorder on the O sublattice in both ZrO_2_ and HfO_2_.

## Conclusion

The performed computations and experiments fill gaps in the available thermodynamic data for pure ZrO_2_ and HfO_2_ at temperatures where the cubic fluorite phase is stable and into the liquid range, thereby facilitating future assessments. The experimental confirmation of thermal expansion and fusion enthalpies validate the accuracy of computational approaches and open the way for further computational studies of the high temperature thermodynamics of more complex systems. Our combined approaches are easy to generalize from HfO_2_ and ZrO_2_ to a broader range of systems. Indeed, we have applied the same combined experimental and computational methods to a wide range of oxides (e.g., Y_2_O_3_^18^, La_2_Zr_2_O_7_^41^, and several rare earth oxides). In addition, the computational method has been employed to study dozens of systems^45^, including oxides^46^, carbides, such as the Hf-Ta-C-N system^47^, and metals^48^.

## Methods

### Computations

We employed first-principles density functional theory^49^ to model HfO_2_ and ZrO_2_. All electronic structures were calculated by the Vienna Ab-initio Simulation Package (VASP)^50^, with the projector-augmented-wave (PAW)^51^ implementation and the generalized gradient approximation (GGA) for exchange-correlation energy, in the form known as Perdew-Burke-Ernzerhof (PBE)^52^. The valence configuration was ([Ar]3*d*^10^)4*s*^2^4*p*^6^4*d*^2^5*s*^2^ with cutoff radius of 1.625 Å for zirconium, ([Kr]4*d*^10^4*f*^14^5*s*^2^) 5*p*^6^5*d*^2^6*s*^2^ with cutoff radius of 1.614 Å for hafnium; for oxygen, the 2 *s* and 2*p* electrons were relaxed with cutoff radius of 0.820 Å. This required a plane-wave basis set with the cutoff energy of 400 eV.

The electronic temperature was accounted for by imposing a Fermi distribution of electrons on the energy level density of states. The electronic temperature was set consistently with the ionic temperature. We used automated *k*-meshes generation with a *k*-point density of 15^3^/Å^−3^ in the Brillouin zone. First-principles molecular dynamics (MD) techniques were utilized to simulate atomic movements and trajectories. The MD simulations were carried out under a constant number of atoms, pressure and temperature condition (*NPT*, isothermal-isobaric ensemble) with a time step around 2fs. The thermostat was conducted under the Nosé-Hoover chain formalism^53,54^. The barostat was realized by adjusting the volume every 80 steps according to average pressure. Although this did not formally generate an isobaric ensemble, this approach has been shown^55^ to provide an effective way to change volume smoothly and to avoid the unphysically large oscillation caused by commonly used barostats. MD simulations were carried out with 90 Zr (or Hf) and 180 O atoms in a periodic cell. Employing periodic boundary conditions is a completely standard way to model extended condensed phases in these types of calculations. The cell size is as large as 16 Å to reduce the finite-size effect. The liquid phase was prepared by heating the solid up to 6000 K (about twice the melting temperature) for 0.5 picoseconds. The liquid is then cooled to the simulation temperature. MD simulations were performed for a sufficiently long time to achieve convergence. The length of MD trajectory varies from 14 to 62 picoseconds, depending on convergence, but generally, 30–50 picoseconds were sufficient. On average, computations took about 25,000 CPU hours per data point, which required around two weeks on 64 cores of a computer cluster. Theoretical X-ray diffraction calculations were carried out using the AFLOW package^56^. MD trajectory was sampled every 80 ionic steps, which formed a set of snapshots that were used to generate X-ray diffraction patterns averaged for the final analysis.

### Experiments

X-ray diffraction (XRD) and calorimetry experiments were performed on polycrystalline ZrO_2_ and HfO_2_ beads, 2–3 mm in diameter, prepared by melting of powders purchased from Alfa Aesar (99.98% or higher metals purity) with a 400 W CO_2_ laser. Samples were first melted into oblate spheroids in a copper hearth, followed by melting in an aerodynamic levitator, as described in detail elsewhere^17^. High temperature XRD experiments were performed with the aerodynamic levitator^57^ at beamline 6-ID-D at the Advanced Photon Source (APS) at Argonne National Laboratory. Diffraction images were collected with a Perkin Elmer XRD1621 amorphous silicon detector in transmission through the upper part of laser heated beads freely rotating in oxygen flow through a levitator nozzle. The X-ray beam (λ = 0.12359(7) Å) was collimated to 0.5 mm wide, 0.2 mm tall rectangular shape. All images were recorded as a sum of 120 0.1 s exposures. The diffraction images at room temperature with the laser off were recorded first; then the sample was heated by a 400 W CO_2_ laser in 50–100 °C increments as monitored with a Chino IR-CAS8CS pyrometer with 1 mm spot size set to 0.92 emissivity and 0.85 window transmission corrections. Image calibration, integration, and sequential Pawley and Rietveld refinements of XRD patterns were performed with the GSAS-II software^58^, backgrounds were fitted manually for each pattern and were not refined. NIST CeO_2_ SRM674b powder standard was used to calibrate detector tilt and rotation angles, beam center position and sample to detector distance (1036.2 mm). Unit cell parameters for laser melted monoclinic ZrO_2,_ and HfO_2_ were refined using conventional powder XRD with internal NIST Si640C standard and Bruker D8 instrument. In a sequential refinement of high temperature patterns, sample displacement was refined for each sample bead at room temperature from calibrated cell parameter and fixed for all refinements of high temperature patterns. In Rietveld refinements, sample absorption and oxygen occupancy were not refined to avoid correlation with atomic displacement parameters. Fusion enthalpies for ZrO_2_ and HfO_2_ were measured using drop and catch calorimetry. The technique and apparatus were described in detail elsewhere^17,18^. Schematic diagrams and photographs are provided in Supplementary Information together with data from all experiments.

## Electronic supplementary material

Supplementary information
