# Supplementary material for: Combined computational and experimental investigation of high temperature thermodynamics and structure of cubic ZrO2 and HfO2
Source: Sci Rep. 2018 Oct 8;8:14962. doi: 10.1038/s41598-018-32848-7 (PMC6175917; doi:10.1038/s41598-018-32848-7)
Supplement: Supplementary file 1 — Supplementary information [file 41598_2018_32848_MOESM1_ESM.docx]

Supplementary information for the manuscript:

**Combined computational and experimental investigation of high temperature thermodynamics and structure of cubic ZrO_2_ and HfO_2_**

Qi-Jun Hong^1^ , Sergey V. Ushakov ^2^, Denys Kapush^2^, Chris J. Benmore^3^, Richard J.K. Weber^3,4^, Axel van de Walle^1^ & Alexandra  Navrotsky^2^

^1^School of Engineering, Brown University, Providence, RI 02912, USA

^2^Peter A. Rock Thermochemistry Laboratory and NEAT ORU, University of California Davis, Davis, CA 95616, USA

^3^X-ray Science Division, Advanced Photon Source, Argonne National Laboratory, 9700 S. Cass Avenue, Lemont, IL 60439, USA

^4^Materials Development, Inc., 3090 Daniels Court, Arlington Heights, IL 60004, USA

**Table of contents:**

Table S1. Results of Pawley refinement of X-ray diffraction patterns with cubic ZrO_2_.

Figure S1. Unit cell parameter for cubic ZrO_2_ plotted *vs.* collection sequence and *vs.* calibrated temperature of diffracted volume.

Figure S2. Volume of tetragonal ZrO_2_ at tetragonal-cubic transition temperature from this work compared with values in ICSD database.

Table S2. Results of Rietveld refinement of selected high temperature X-ray diffraction patterns of cubic ZrO_2_.

Figure S3. Diffraction image with highlighted integration area and Rietveld refinement plot of cubic ZrO_2_.

Table S3. Results of Pawley refinement of X-ray diffraction patterns of cubic HfO_2_.

Table S4. Results of Rietveld refinement of selected X-ray diffraction patterns of cubic HfO_2_.

Figure S4. Isotropic atomic displacement parameters from Rietveld refinement of X-ray data compared with results of *ab initio* MD simulations.

Table S5. Average diffusion rates in cubic and liquid ZrO_2_ and HfO_2_ from *ab initio* MD computations.

Figure S5. Schematics and photograph of Drop-and-Catch calorimeter system and heat flow traces for HfO_2_ and ZrO_2_ caught in liquid and solid state.

Table S6. Drop-and-Catch experiments on ZrO_2_ and HfO_2_ levitated in argon flow.

Table S7. Drop-and-Catch experiments on ZrO_2_ and HfO_2_ levitated in oxygen flow.

Figure S6. Drop-and-Catch experiments on ZrO_2_ and HfO_2_ levitated in oxygen flow.

Figure S7: Diffusion of oxygen and zirconium atoms in cubic and liquid zirconia at various temperatures from computer simulations. (*x)* is the change in atomic position in MD trajectory, and *p*(*x*) is the probability distribution.

Figure S8: Diffusion of oxygen and hafnium atoms in cubic and liquid hafnia at various temperatures from computer simulations. (*x)* is the change in atomic position in MD trajectory, and *p*(*x*) is the probability distribution.

Table S1. Results of Pawley refinement^†^ of X-ray diffraction patterns with cubic ZrO_2_

| APS | ^‡^T_s_ | T_v_ | *R*_wp_ | Tetragonal | | | Cubic | |
| --- | --- | --- | --- | --- | --- | --- | --- | --- |
| File ID | °C | °C |  | *a,* Å | c*,* Å | Vol, Å^3^ | *a,* Å | Vol, Å^3^ |
| 00369 | 2700 | 2311 | 3.5 | 3.6888(1) | 5.3372(2) | 72.63(1) | 5.2653(1) | 145.97(1) |
| 03370 |  |  | 3.6 | 3.6899(1) | 5.3367(2) | 72.66(1) | 5.2651(1) | 145.96(1) |
| 03371 |  |  | 3.6 | 3.6894(1) | 5.3367(2) | 72.64(1) | 5.2651(1) | 145.96(1) |
| 03372 | 2750 | 2411 | 5.9 | 3.6912(1) | 5.3373(2) | 72.72(1) | 5.2688(1) | 146.27(1) |
| 03373 |  |  | 4.2 | 3.6909(1) | 5.3349(3) | 72.67(1) | 5.2699(1) | 146.36(1) |
| 03374 |  |  | 4.1 | 3.6921(1) | 5.3369(7) | 72.75(1) | 5.2710(1) | 146.44(1) |
| 03375 | 2800 | 2511 | 9.2 |  |  |  | 5.2747(1) | 146.75(1) |
| 03376 |  |  | 7.9 |  |  |  | 5.2767(1) | 146.92(1) |
| 03377 |  |  | 7.1 |  |  |  | 5.2778(1) | 147.01(1) |
| 03378 | 2850 | 2610 | 5.4 |  |  |  | 5.2848(1) | 147.60(1) |
| 03379 |  |  | 7.7 |  |  |  | 5.2851(1) | 147.62(1) |
| 03380 |  |  | 7.1 |  |  |  | 5.2870(1) | 147.78(1) |
| 03381 | 2900 | 2710 | 4.5 |  |  |  | 5.2920(1) | 148.20(1) |
| 03382 |  |  | 4.7 |  |  |  | 5.2917(2) | 148.18(2) |
| 03383 |  |  | 4.1 |  |  |  | 5.2921(1) | 148.21(1) |

^†^55.27 mg bead (oblate spheroid 2.78 x2.5 mm) leviated in oxygen flow; room tempeature unit cell for laser melted m-ZrO_2_ a = 5.1483(3) Å, b = 5.2118(3) Å, c = 5.3132(3) Å, β = 99.205(2)°

^‡^Surface temperature from Chino IRCAS8CS pyrometer (1 mm spot size, 0.92 emissivity and 0.85 window transmission correction)


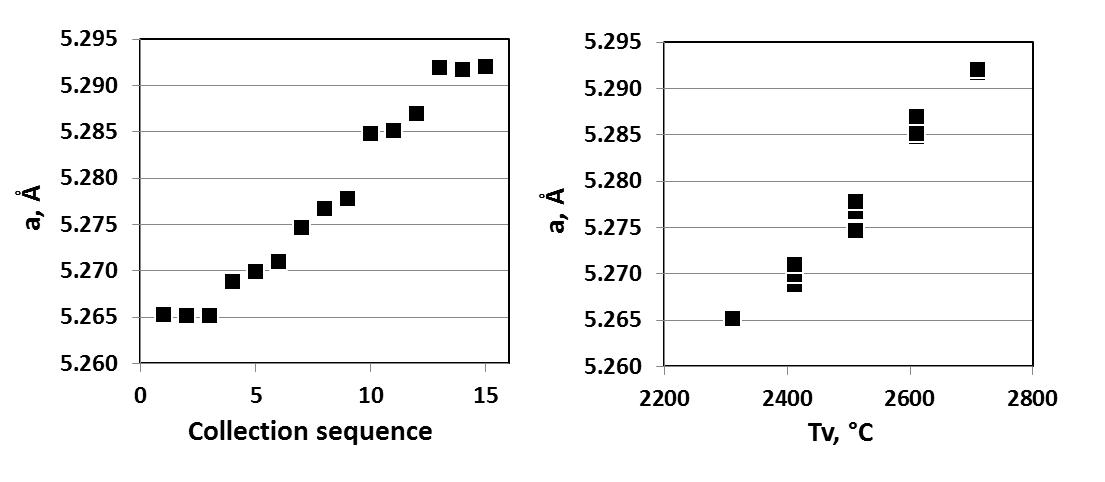


Figure S1. Unit cell parameter for cubic ZrO_2_ plotted *vs.* collection sequence and *vs.* calibrated temperature of diffracted volume. The results of Pawley refinement of unit cells for each pattern are listed in Table S1. The smallest and largest values correspond to XRD patterns where cubic ZrO_2_ is coexist with tetragonal phase (2310 °C) and with melt (2710 °C).

| **ICSD #** | T, °C | T-ZrO_2_  Vol, Å^3^ |
| --- | --- | --- |
| 70014 | 22 | 66.72 |
| 72950 | 297 | 67.78 |
| 72951 | 497 | 68.32 |
| 72706 | 700 | 68.83 |
| 72952 | 787 | 69.08 |
| 72953 | 927 | 69.39 |
| 72702 | 947 | 69.71 |
| 72703 | 997 | 69.44 |
| 93125 | 1000 | 69.97 |
| 72704 | 1107 | 69.7 |
| 72954 | 1127 | 69.92 |
| 72705 | 1177 | 69.88 |
| 93126 | 1200 | 70.26 |
| 9993 | 1200 | 69.83 |
| 23928 | 1250 | 69.83 |
| 72706 | 1277 | 70.12 |
| 72707 | 1327 | 70.23 |
| 647692 | 1393 | 70.61 |
| 72708 | 1627 | 70.97 |
| **This work** | **2311** | **72.68** |

Figure S2. Volume of tetragonal ZrO_2_ at tetragonal-cubic transition temperature from this work compared with values in ICSD database.

Table S2. Results of Rietveld refinement† of selected high temperature X-ray diffraction patterns of cubic ZrO_2_. Atomic displacement parameters (*U*_iso_) from ab initio MD calculations are shown for comparison.

| APS | T_s_ | T_v_ | *R*_wp_ | GOF | *a,* Å | Vol, Å^3^ | 100·*U*_iso_ , Å^2^ | |
| --- | --- | --- | --- | --- | --- | --- | --- | --- |
| File ID | °C | °C | _%_ |  |  |  | Zr | O |
| 03377 | 2800 | 2511 | 3.4 | 5.6 | 5.2786(1) | 147.08(1) | 5.2(1) | 11.0(2) |
| 03380 | 2850 | 2610 | 3.9 | 6.5 | 5.2876(1) | 147.83(1) | 6.0(1) | 8.1(3) |
| 03383 | 2900 | 2710 | 2.0 | 3.3 | 5.2923(1) | 148.23(1) | 5.3(1) | 15.1(5) |
| ***Ab initio* MD results** | | | | | | | | |
|  |  | **2527** |  |  |  |  | **3.3(1)** | **19(1)** |
|  |  | **2627** |  |  |  |  | **3.9(1)** | **24(1)** |
|  |  | **2727** |  |  |  |  | **4.9(1)** | **29(1)** |

†Limits were set to 3-10 ° 2 theta; sample absorption fixed to 3.63 µr for *U*_iso_ refinement, atomic fractions were not refined.


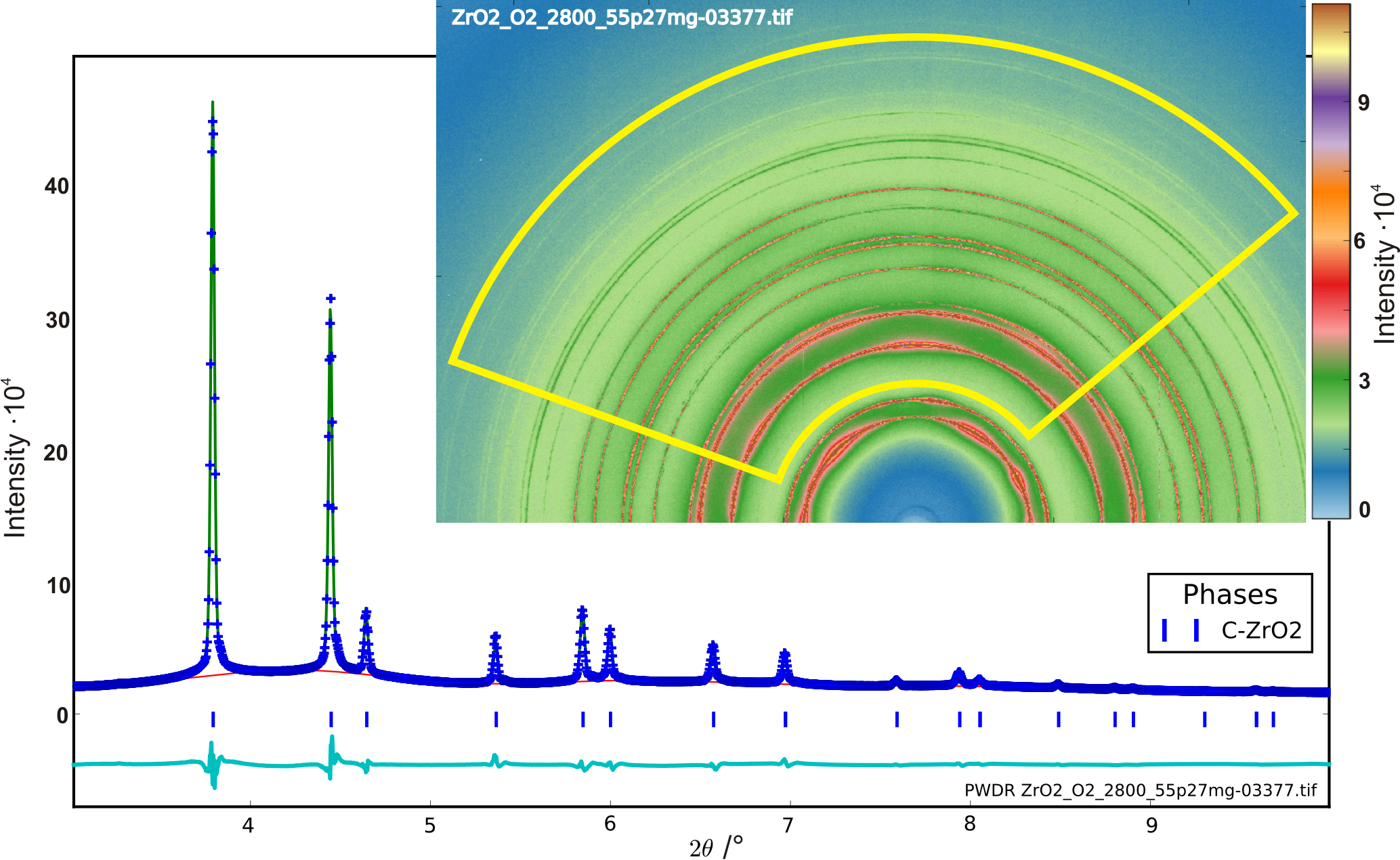


Figure S3. Diffraction image with highlighted integration area and Rietveld refinement plot of cubic ZrO_2_ (Tv = 2511 °C, APS file ID 03377), see Table S2 for refinement results and metrics).

Table S3. Results of Pawley refinement^†^ of X-ray diffraction patterns of cubic HfO_2_.

| APS | ^‡^T_s_ | T_v_ | | *R*_wp_ | Tetragonal | | | Cubic | |
| --- | --- | --- | --- | --- | --- | --- | --- | --- | --- |
| File ID | °C | °C | |  | *a,* Å | c*,* Å | Vol, Å^3^ | *a,* Å | Vol, Å^3^ |
| 02310 | 2500 | 2530 | 3.19 | | 3.6677(2) | 5.3251(5) | 71.63(1) | 5.2461(1) | 144.38(1) |
| 02313 | 2600 | 2572 | 2.89 | | 3.6695(2) | 5.3246(6) | 71.70(1) | 5.2482(1) | 144.56(1) |
| 02316 | 2700 | 2613 | 2.98 | | 3.6688(4) | 5.328(1) | 71.72(2) | 5.2509(2) | 144.78(1) |
| 02319 | 2800 | 2655 | 2.70 | | 3.6710(6) | 5.328(2) | 71.80(3) | 5.2534(1) | 144.98(1) |
| 02322 | 2900 | 2696 | 3.34 | |  |  |  | 5.2549(2) | 145.11(1) |
| 02325 | 2950 | 2717 | 2.74 | |  |  |  | 5.2560(1) | 145.20(1) |
| 02328 | 3000 | 2738 | 2.50 | |  |  |  | 5.2586(1) | 145.41(1) |
| 02331 | 3050 | 2758 | 2.67 | |  |  |  | 5.2589(1) | 145.44(1) |
| 02334 | 3100 | 2779 | 3.05 | |  |  |  | 5.2615(2) | 145.65(2) |
| 02337 | 3150 | 2800 | 3.44 | |  |  |  | 5.2646(5) | 145.91(4) |

^†^120.65 mg bead (oblate spheroid 3.0 x 2.6 mm) leviated in oxygen flow; room tempeature unit cell for laser melted m-HfO_2_ *a =* 5.1172(2), *b =* 5.1808(2), *c =* 5.2860(2), c = 99.250(2) º

^‡^Surface temperature from Chino IRCAS8CS pyrometer (1 mm spot size, 0.92 emissivity and 0.85 window transmission correction)

Table S4. Results of Rietveld refinement† of selected diffraction patterns of cubic HfO_2_. Atomic displacement parameters (*U*_iso_) from ab initio MD calculations are shown for comparison.

| APS | T_s_ | T_v_ | *R*_wp_ | GOF | *a,* Å | Vol, Å^3^ | 100·*U*_iso_  Hf O | |
| --- | --- | --- | --- | --- | --- | --- | --- | --- |
| File ID | °C | °C | _%_ |  |  |  |  |  |
| 02322 | 2900 | 2696 | 4.5 | 1.6 | 5.2544(2) | 145.07(1) | 4.6(1) | 3.2(6) |
| 02325 | 2950 | 2717 | 3.2 | 1.1 | 5.2555(1) | 145.16(1) | 5.3(1) | 6.9(8) |
| 02331 | 3050 | 2758 | 3.2 | 1.1 | 5.2577(2) | 145.34(1) | 5.4(2) | 3.3(7) |
| 02334 | 3100 | 2779 | 2.9 | 1.0 | 5.2602(2) | 145.55(2) | 5.0(2) | 7(1) |
| ***Ab initio* MD results** | | | | | | | | |
|  |  | **2527** |  |  |  |  | **3.3(1)** | **4.8(6)** |
|  |  | **2627** |  |  |  |  | **3.7(1)** | **5.6(7)** |
|  |  | **2727** |  |  |  |  | **3.7(1)** | **7.7(9)** |

†Limits were set to 3-7 ° 2 theta; sample absorption fixed to 5.6 µr for *U*_iso_ refinement, atomic fractions were not refined.


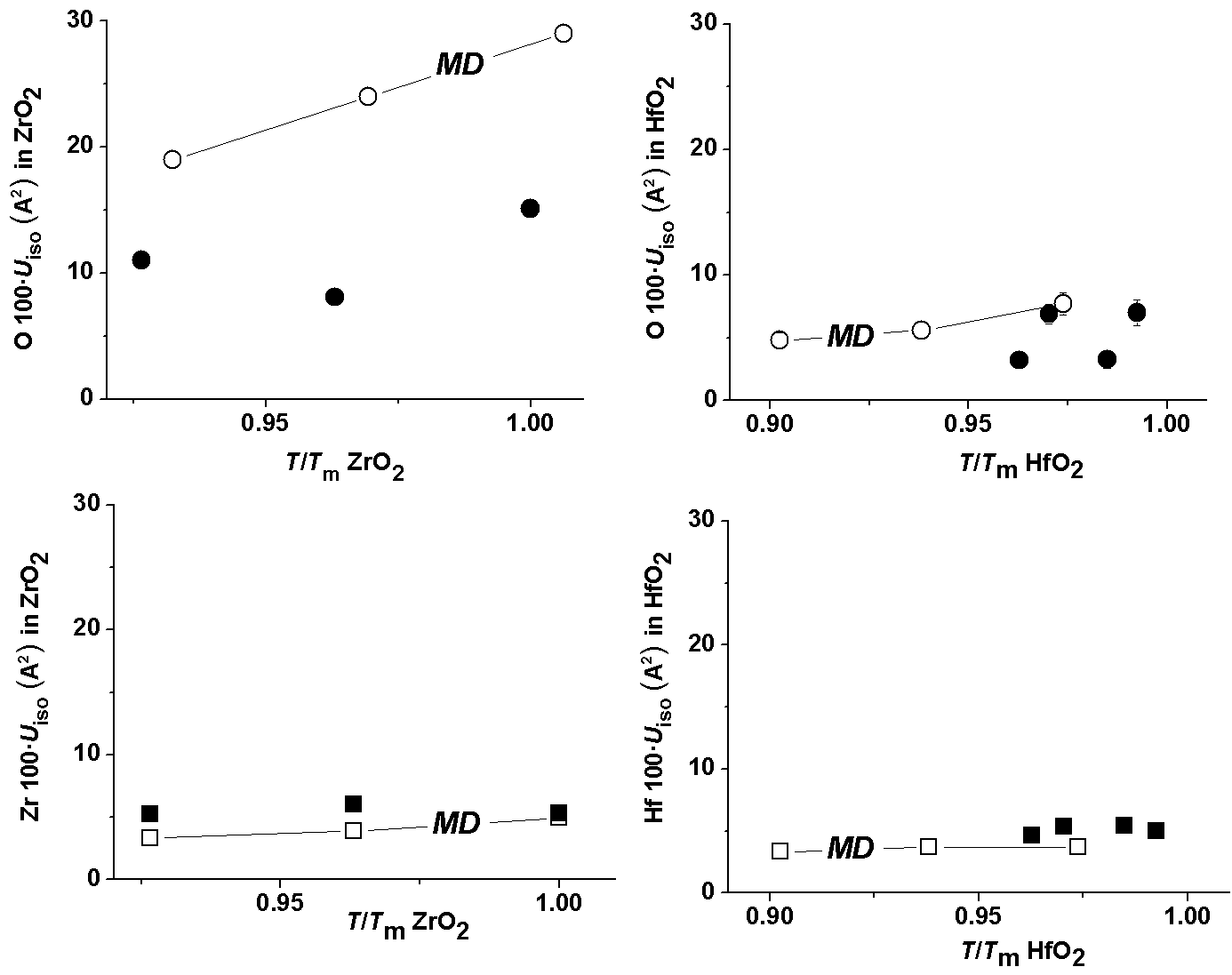


Figure S4. Isotropic atomic displacement parameters (100·*U*_iso_, Å^2^) for Zr, Hf and Oxygen versus T/T_m_ (T_m_ – melting temperature) from Rietveld refinement of X-ray data (solid symbols) compared with results of *Ab initio* MD simulations (open symbols).

Table S5. Diffusion coefficients in cubic and liquid ZrO_2_ and HfO_2_ from *ab initio* MD computations.

| Phase | T,°C | ZrO_2­_ | | HfO_2_ | |
| --- | --- | --- | --- | --- | --- |
|  |  | O, ×10^-5^ cm^2^/s | Zr, ×10^-5^ cm^2^/s | O, ×10^-5^ cm^2^/s | Hf, ×10^-5^ cm^2^/s |
| Cubic | 2327 | 0.7±0.3 | 0.05±0.02 | (0.08±0.04) † | (0.02±0.01)† |
|  | 2527 | 2.1±0.2 | 0.04±0.01 | 0.8±0.2 | 0.01±0.01 |
|  | 2627 | 2.6±0.3 | 0.05±0.02 | 1.2±0.2 | 0.01±0.01 |
|  | 2727 | 3.2±0.3 | 0.07±0.01 | 1.5±0.1 | 0.03±0.01 |
| Liquid | 2827 | 8.6±0.3 | 3.7±0.3 | 5.2±0.2 | 2.4±0.1 |

† Computed diffusion rates for Hf and O in cubic HfO_2_ at 2327 °C are below accepted tetragonal-cubic transformation temperature (2530 °C)


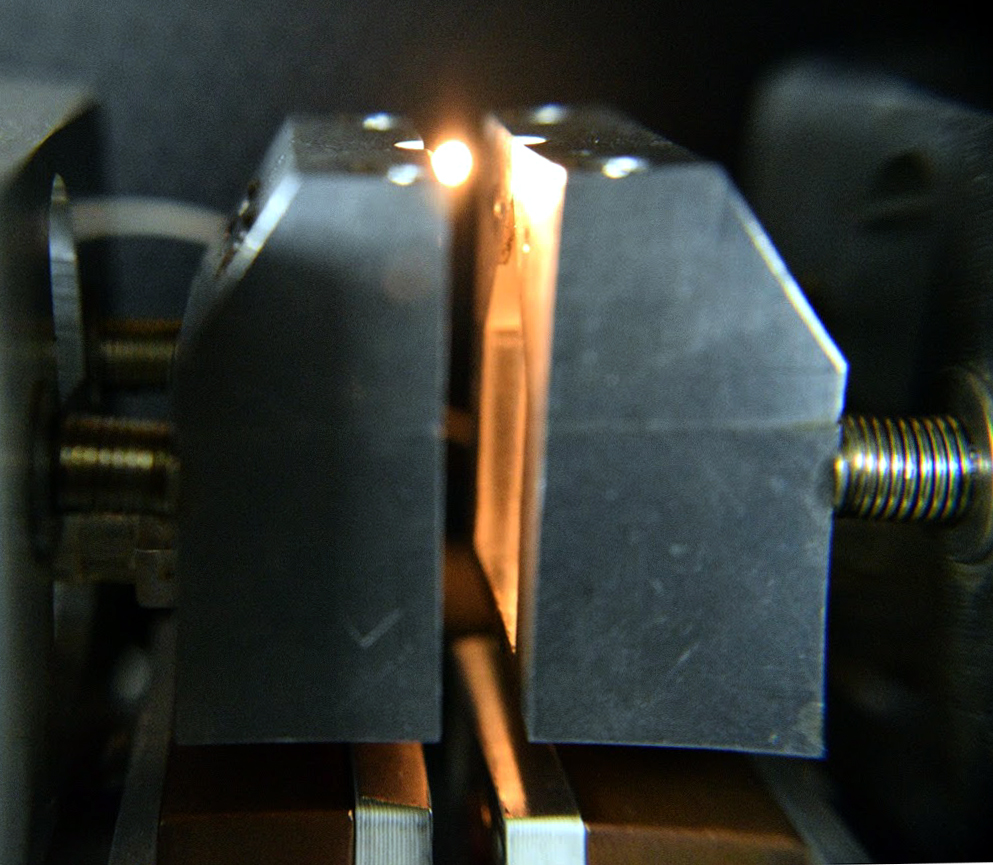




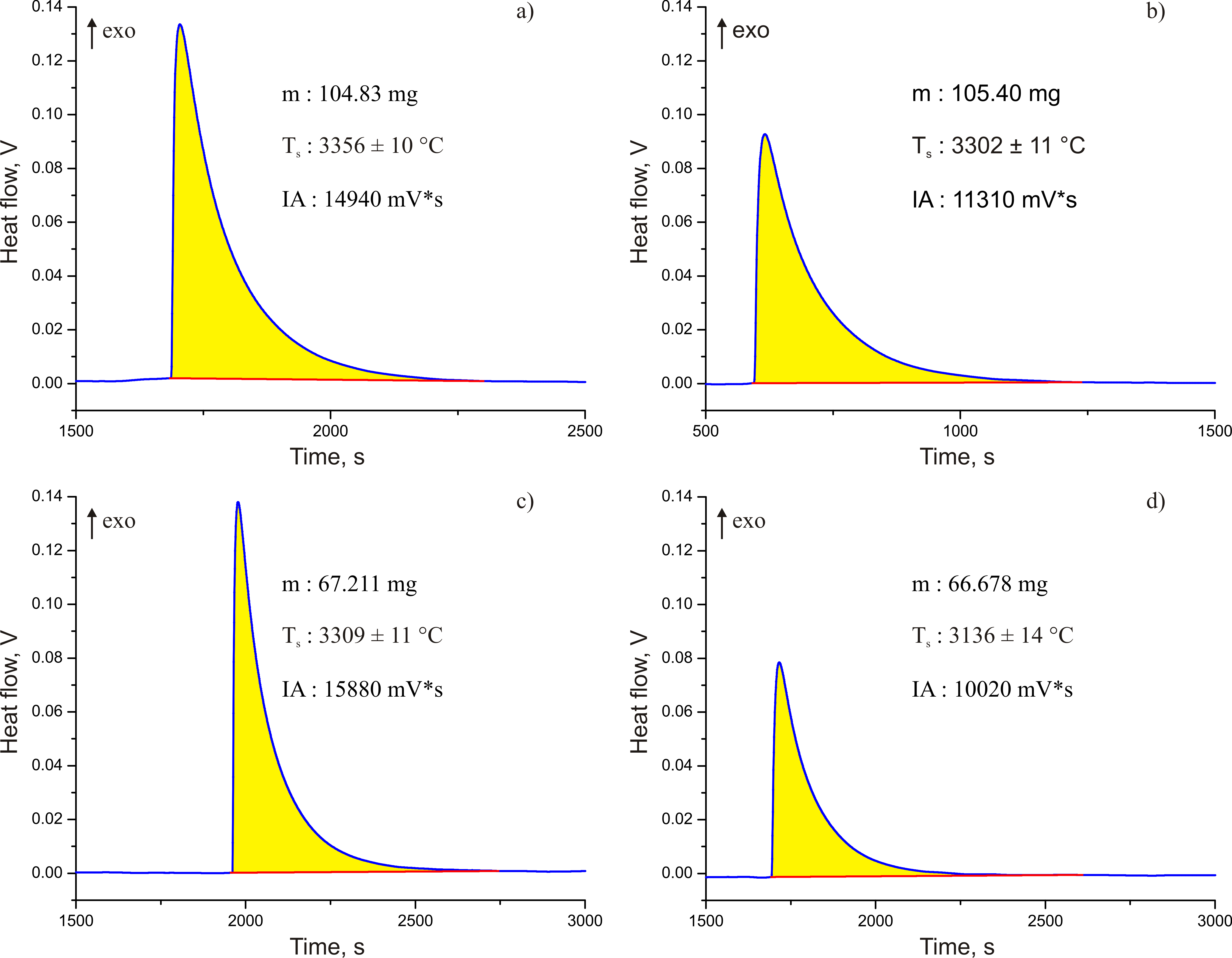


Figure S5. Schematics and photograph of Drop-and-Catch calorimeter system and heat flow traces for HfO_2_ and ZrO_2_ caught in liquid and solid state. (a,b – HfO_2_, c,d – ZrO_2_).

Table S6. Drop-and-Catch experiments^§^ on ZrO_2_ and HfO_2_ levitated in argon flow.

| # | **ZrO_2_**  (mg) | T_s_^†^  (°C) | ΔH (T_s_-25 °C)  (kJ/mol) | # | **HfO_2_**  (mg) | T_s_^†^  (°C) | ΔH (T_s_-25 °C)  (kJ/mol) |
| --- | --- | --- | --- | --- | --- | --- | --- |
| 1 | 62.03 | 2674 ±10 | 133.0 ±2.7 | 1 | 118.33 | 2470 ±8 | 105.0 ±2.1 |
| 2 | 62.02 | 2712 ±11 | 123.9 ±2.5 | 2 | 118.61 | 2653 ±10 | 120.4 ±2.4 |
| 3 | 69.44 | 2802 ±12 | 132.7 ±2.7 | 3 | 118.56 | 2723 ±12 | 121.2 ±2.4 |
| 4 | 60.95 | 2940 ±14 | 135.2 ±2.7 | 4 | 118.41 | 2756 ±11 | 132.9 ±2.7 |
| 5 | 61.95 | 3022 ±13 | 158.8 ±3.2 | 5 | 118.45 | 2823 ±12 | 138.2 ±2.8 |
| 6 | 65.89 | 3122 ±14 | 168.0 ±3.4 | 6 | 116.19 | 2924 ±17 | 146.9 ±2.9 |
| 7 | 65.61 | 3229 ±15 | 223.2 ±4.5 | 7 | 105.66 | 3067 ±14 | 161.6 ±3.2 |
| 8 | 62.36 | 3267 ±12 | 228.7 ±4.6 | 8 | 105.64 | 3113 ±12 | 169.5 ±3.4 |
| 9 | 61.64 | 3332 ±13 | 232.4 ±4.6 | 9 | 105.58 | 3211 ±12 | 175.2 ±3.5 |
| 10 | 60.63 | 3468 ±14 | 252.1 ±5.0 | 10 | 105.40 | 3302 ±11 | 187.3 ±3.8 |
|  |  |  |  | 11 | 104.83 | 3356 ±10 | 248.8 ±5.0 |
|  |  |  |  | 12 | 109.86 | 3530 ±8 | 254.8 ±5.1 |
|  |  |  |  | 13 | 100.00 | 3733 ±6 | 264.8 ±5.3 |
|  |  |  |  | 14 | 100.52 | 3756 ±5 | 267.9 ±5.4 |

^§^Catch delay 104 ms, † Surface temperature measured by FAR spectropyrometer

Table S7. Drop-and-Catch experiments on ZrO_2_ and HfO_2_ levitated in oxygen flow.

| # | **ZrO_2_**  (mg) | T_s_†  (°C) | ΔH (T_s_-25 °C)  (kJ/mol) | # | **HfO_2_**  (mg) | T_s_†  (°C) | ΔH (T_s_-25 °C)  (kJ/mol) |
| --- | --- | --- | --- | --- | --- | --- | --- |
| 1 | 67.74 | 2417 ±11 | 116.6 ±2.3 | 1 | 118.68 | 2589 ±10 | 127.2 |
| 2 | 67.73 | 2497 ±14 | 126.7 ±2.5 | 2 | 118.61 | 2653 ±10 | 120.4 |
| 3 | 67.72 | 2593 ±13 | 116.4 ±2.3 | 3 | 118.56 | 2723 ±12 | 121.2 |
| 4 | 67.52 | 2623 ±13 | 125.4 ±2.5 | 4 | 118.41 | 2756 ±11 | 132.9 |
| 5 | 67.20 | 2798 ±13 | 136.4 ±2.7 | 5 | 118.45 | 2823 ±12 | 138.2 |
| 6 | 67.18 | 2876 ±12 | 140.0 ±2.8 | 6 | 116.19 | 2924 ±17 | 146.9 |
| 7 | 67.10 | 2957 ±12 | 146.9 ±2.9 | 7 | 115.99 | 3060 ±13 | 147.8 |
| 8 | 66.68 | 3136 ±14 | 153.5 ±3.1 | 8 | 108.83 | 3152 ±12 | 154.8 |
| 9 | 72.46 | 3180 ±11 | 174.9 ±3.5 | 9 | 101.38 | 3262 ±12 | 160.9 |
| 10 | 68.31 | 3204 ±12 | 189.4 ±3.8 | 10 | 118.33 | 3326 ±10 | 175.4 |
| 11 | 70.12 | 3263 ±11 | 222.2 ±4.4 | 11 | 101.26 | 3418 ±10 | 201.3 |
| 12 | 67.21 | 3309 ±11 | 241.4 ±4.8 | 12 | 98.38 | 3487 ±8 | 202.2 |
| 13 | 67.05 | 3405 ±8 | 247.0 ±4.9 | 13 | 97.76 | 3660 ±7 | 210.9 |
| 14 | 75.54 | 3448 ±8 | 251.0 ±5.0 | 14 | 91.31 | 3710 ±7 | 222.3 |
| 15 | 64.44 | 3618 ±6 | 255.4 ±5.1 | 15 | 82.68 | 3832 ±7 | 254.6 |

_
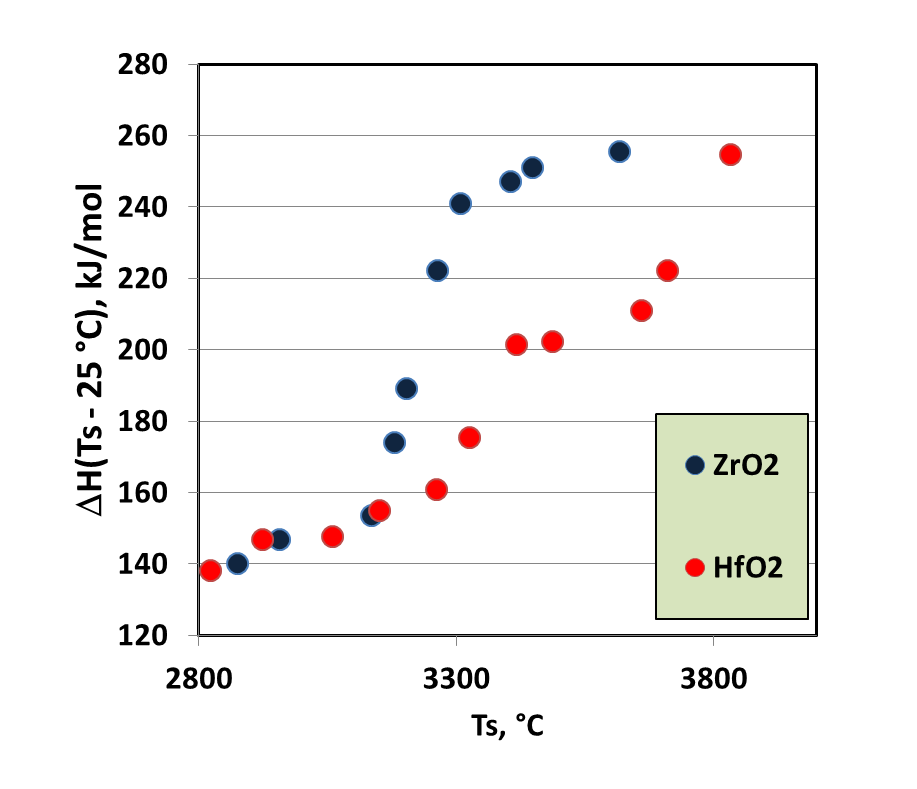
_

Figure S6. Drop-and-Catch experiments on ZrO_2_ and HfO_2_ levitated in oxygen flow. Catch delay 104 ms, Ts - surface temperature measured by spectropyrometer.

Figure S7: Diffusion of oxygen and zirconium atoms in cubic and liquid zirconia at various temperatures from computer simulations. *x* is change in atomic position in MD trajectory, and *p(x)* is probability distribution.

Figure S8: Diffusion of oxygen and hafnium atoms in cubic and liquid hafnia at various temperatures from computer simulations. *x* is change in atomic position in MD trajectory, and *p(x)* is probability distribution.
